# Experiences of Female Rugby Union Players and Practitioners in Rehabilitation Following a Shoulder Injury

**DOI:** 10.3390/sports13060166

**Published:** 2025-05-28

**Authors:** Caroline Sarah White, Paul Garner, Ian Horsley, Andrew Soundy

**Affiliations:** 1 School of Sport, Exercise and Rehabilitation, Graduate School of Sport and Professional Practice, University of Birmingham, Edgbaston Campus, Birmingham B15 2TT, UK; 2 UK Sports Institute, 299 Alan Turing Way, Manchester M11 3BS, UK

**Keywords:** qualitative, care, shoulder, rugby, female-athlete

## Abstract

Background: Little is known about the perspectives of elite female rugby union players and practitioners towards rehabilitation and return to play (RTP) following shoulder injuries and how to manage these complex injuries. Methods: An interpretive hermeneutic phenomenology study was undertaken within a subtle realist paradigmatic view. Twenty semi-structured interviews were conducted with elite female rugby union players and practitioners working in elite female rugby union. Data were analysed using reflective thematic analysis. Results: Four themes emerged from the data: (1) growth of the women’s game, which involved several influences on the support and resources players received; (2) different viewpoints between players and practitioners concerning injury reporting and objective testing; (3) threats to identity, a player-only generated theme centred around the risk to their career following a significant injury and the isolation they faced; and (4) RTP, exploring strategies and influences to players’ and practitioners’ confidence in the RTP process following a shoulder injury. Conclusions: Practitioners should consider the non-physical factors during the rehabilitation process and the theory of care concept in supporting and collaborating with female rugby union players following a shoulder injury.

## 1. Introduction

Rugby Union is a fast-growing invasion sport, notably for elite female athletes. It involves contact events, such as scrummaging, mauls and tackling, with high-intensity running [1]. A tackle event is when there is direct contact between an opponent and the ball carrier; they are either tackling as the opponent or being tackled as the ball carrier [2]. The tackle event has been highlighted as the primary cause of injuries in women’s rugby [1], and with the rise in frequency of physical collisions in the women’s game, this could escalate adverse stress on the body and the potential risk of injury [3]. Injuries are a part of the sport, and the return to sport (RTS) process can be difficult to navigate for both the athlete and practitioner [4]. Concussion and knee ligament injuries are the primary reported injuries within the women’s game [1]. However, there has been little investigation into upper limb injuries. It has been reported that shoulder injuries have a high reoccurrence rate, with athletes reporting instability despite surgical reconstruction [5]. In addition to physical symptoms, patients have also reported emotional distress [6], which has harmed their prognosis and ability to return to play [7]. In a study by Chester et al. [8] that investigated various shoulder injuries in the general population, they found that the significance of the diagnosis did not correlate consistently with the prognosis. Instead, other factors, such as psychosocial issues, had a greater influence on the rehabilitation process, which suggests an urgent need to consider the return to play journey from a more holistic perspective rather than from a biological perspective. Given this imperative, it is important to acknowledge the myriad factors associated with returning to full-blown competitive rugby after having sustained a shoulder injury. The 2022 Bern Consensus [9] highlighted this complexity, noting the lack of agreement on the key stages of rehabilitation and the ambiguity surrounding outcome measures used to assess an athlete’s progress. There is a lack of understanding around how to best manage environmental and psychosocial factors alongside the biological healing process. Therefore, it is our contention that by investigating these issues from the perspective of the multiple stakeholders involved in the rehabilitation process, we can promote a more person-centred approach [10] to supporting players in their return to play from shoulder injury in women’s rugby.

## 2. Materials and Methods

### 2.1. Qualitative Approach and Research Paradigm Study Design

The philosophical approach for this study is subtle realism. Subtle realism is a philosophical stance commonly employed in qualitative research, which seeks to balance absolute objectivity and complete subjectivity. It recognises that, while researchers cannot entirely eliminate their biases, they can aim to represent a common or consistent version of reality identified by the qualitative researcher’s interpretation to achieve a credible and trustworthy interpretation of data. This approach was introduced by Hammersley [11] as a response to the debates around positivism and constructivism, offering a middle ground. Subtle realists are situated to claim generalisation from single to multiple cases, termed naturalistic generalisation [12]. This identifies that elements of the findings can be recognised across different settings, but do not claim to represent ‘the truth’. This approach was used in conjunction with interpretive hermeneutic phenomenology for this research. Interpretive hermeneutic phenomenology explores the athletes’ and practitioners’ lived experiences to give them meaning. Using them in combination, this research sought to explore lived experiences while maintaining a belief in an external reality. This approach honours researchers’ interpretations of the subjective meaning of experience.

### 2.2. Positionality

None of the authors are ex/professional rugby players. Two authors (CW and IH) have experience as physiotherapy practitioners in elite women’s rugby union. The other two authors (AS and PG) have extensive experience in sports qualitative research. The lead author (CW) is a female experienced chartered physiotherapist who has worked in elite sports for over 18 years in the United Kingdom, including rugby union. The lead researcher conducted all the interviews and asked the participants critical questions (see Appendix A). This enabled the lead researcher to bring knowledge and insight from the sport as a clinician and researcher to capture rich data. The members of the collective authorship team, utilising their experience in research and sport, worked as critical friends, exploring the data captured to help represent the viewpoints of the players and practitioners.

### 2.3. Participants, Sampling and Sample Size

The inclusion criteria for players to participate in this study was, female rugby players who had a minimum of one season of playing within the professional rugby union club league and/or representing at the international level were recruited for focus groups, with players who had incurred within the last three years a shoulder injury that kept them out from playing for a minimum of six weeks and to have successfully returned to play were invited to individual interviews. The inclusion criteria for support staff to participate were practitioners from strength and conditioning, doctors and physiotherapists who work within the professional club league or internationally with the 15 s or 7 s. All participants were over the age of eighteen.

Over the nine interviews, four focus groups and five individual interviews, 20 people were interviewed: four physiotherapists, one doctor, four strength and conditioning coaches and 11 players. Five males from within the practitioner group and four female practitioners were interviewed. All participants were based within the United Kingdom.

Focus groups were conducted with practitioners and players who had not incurred a 6-week or longer shoulder injury within the last three years. Individual interviews were conducted with elite female rugby players who had a shoulder injury within the last three years from which they had successfully returned.

Using information power, participants were recruited through purposive sampling [13]. The lead author had an established connection to a network of suitable potential participants. The information provided by the participants was rich in detail and highly relevant to the specific nature of the study, which meant fewer participants were needed. After nine interviews, no new categories or codes emerged, indicating data saturation [14].

### 2.4. Data Collection

Before data collection, ethics were obtained from the University of Birmingham—Ethics Number ERN_0856—March 2023. Data was stored according to the University of Birmingham’s research data storage policy on a password-protected computer, and no identifying documents were linked.

The lead author emailed all England Women’s national and PWR-Club medical staff through the Rugby Football Union (RFU) network, requesting them to circulate the advertisement for the study. Each interested participant was contacted by email, informed of the study’s background and asked to sign an informed consent form. Depending on the participant’s availability, interviews were conducted via Zoom between October 2023 and January 2024. Following two pilot interviews, one with players and another with practitioners, the research team drew upon pilot feedback and extant literature on injury prevention and return to sport (RTS) to develop an interview guide (please see Appendix A).

Four focus groups and five individual semi-structured interviews were conducted by the lead author (CW), and no field notes were taken. The interviews lasted 50–70 min, and the mean length was 60 min. Out of the 20 participants, 15 of them were known to the lead author in a professional capacity. However, she had not worked with any of them directly for at least 6 months before data collection began. The study’s inception and conduction were separate from the organisation where the participants and lead author worked. To ensure open, candid discussions, participants were explicitly informed that the study was conducted in a trusted and confidential space, with all responses anonymised to protect their privacy. They had the right not to participate with no consequences and were informed of their withdrawal rights.

### 2.5. Data Analysis

All interviews were audio recorded via Zoom and transcribed verbatim. Transcriptions were analysed using reflexive thematic analysis, which involved the six stages outlined by Braun and Clarke [15]. In stage one, the lead author (CW) familiarised themselves with the data. This included listening to the audio recordings multiple times and reading the transcriptions. Stage two was generating initial codes and breaking the data into meaningful elements. Due to the numerous shared codes between the focus groups and individual interviews and the focus of the analysis on common experiences (as part of the output from subtle realism), researchers decided to combine the data. Stage three was to see what initial themes emerged. Recurring keywords or patterns were highlighted directly in the transcriptions. Stage four was to review the themes and ensure they accurately represented the data (See Supplementary File S1). Given the interpretive activity and epistemological underpinning of this study, we approached trustworthiness by employing a thorough reflexive process involving the second (PG) and fourth (AS) authors, as opposed to any form of member checking, which has been discouraged in contemporary literature [2]. Stage five was defining and naming the themes, identifying key elements and assigning codes to data segments to capture their core message. The codes and quotes they were related to were inserted into an Excel spreadsheet to assist with theme generation. Similar codes were grouped to develop various themes and interpretive stories of the data, with conceptualising, understanding and defining the concepts that emerged from the data. With PG and AS, reflexive discussions were had over the identified themes and sub-themes to develop a rich data reading. Stage six involved writing up the report of findings and insights, drawing upon inductive reasoning to address the overall aims of the project.

### 2.6. Quality of Data

The consolidation criteria for reporting qualitative research (COREQ) checklist [16] were followed. Trustworthiness strategies that are consistent with subtle realism were selected [17], as well as being recognised by review evidence as important items for quality [18]. This included credibility through peer review, data collection up to saturation, and respondent validation. Transferability involved having maximum variation and thick description. Dependability and comfortability involved having an audit trail and reflexivity throughout the process.

## 3. Results and Discussion

From the data, four main themes emerged (please see Table 1: (1) growth of the women’s game, (2) different viewpoints between athletes and practitioners, (3) threat to identity, and (4) return to play (RTP)). 

### 3.1. Theme 1: Growth of the Women’s Game

This theme captures the perception of gender-related gaps in the provision of services to elite women rugby players following the recent growth of the game. The gaps are identified within the sub-themes as tackle technique, strength training, pitch surfaces and turnover of staff. These broad factors have been identified in previous research, such as a Delphi study in the Women’s Rugby League by Scantlebury et al. [19], which found that a lack of facilities, pitches, and qualified staff provision increases injury risk.

#### 3.1.1. Tackle Technique

The first sub-theme focussed on a specific element that has evolved as the women’s game has grown. Most participants—players and practitioners—raised concerns about tackle technique, with a feeling that there is a shorter timeframe between players starting to play rugby and performing at a professional level. One participant reflected on this short timeframe:

*“We can’t just get away with having young girls come through and just be like, okay, well, they can tackle that’s fine…is it a good tackle?”*

(P15)

This raised concerns about a potential lack of exposure to regular coaching on tackle technique linked to limited opportunities to develop contact skills. They felt that when players were fatigued, the tackle technique was one of the first things to deteriorate, which can increase the likelihood of injury. Due to increased tackle frequency [2], World Rugby has been investigating ways to manage injuries related to tackle events [20]. Coaching players to effectively and safely tackle reduces their injury risk [21], which places real importance on the coaching approach to which female athletes are exposed. A global survey by Dane et al. [22] explored tackle training and knowledge in female coaches. They found that only 17% of the 60 coaches surveyed adopted female-specific approaches, which included breast impact, managing a diverse age range and approaches to confidence building. This was despite 70% of coaches having a high intention to adopt these practices. This could have been influenced by having engaged with previous coach development that was designed for the men’s game, with little support to adapt and translate content for female athletes. Although it is encouraging how many coaches want to improve their female-specific coaching, further support is required to assist them with delivery, which could be addressed by using mentors and promoting female-specific coach education sessions.

#### 3.1.2. Strength Training

This sub-theme shared similarities with the tackle technique sub-theme, which other factors have influenced. Four practitioners discussed that there was no formal framework to follow within the women’s game. The English RFU intends to develop a game-wide, strength-based curriculum to support female players’ physical development at the club and international levels. Participant 12 spoke about their plans:

*“Performance and development, a lot of it sits in development space, basic strength bullet-proofing (injury prevention). But the link is around some collision dominance, taking space, making space…the club practitioners will then have a really easy framework to refer back to rather than having to build their own”.*

(P12)

This links back to the previous sub-theme of tackle technique. S&C coaches have the desire to support female athletes, specifically in the gym, but they lack education and understanding of menstrual health, female physiology and psychology [23]. One practitioner noticed an improvement in the player’s engagement in the strength sessions. This was identified when the player understood a connection between the work they did in the gym and their performance on the rugby field. As they remarked,

*“really good wins when it came to just general high-level understanding of why we would do an upper body lift from a strength perspective”.*

(P15)

Players and practitioners both identified that strength development was an area that needed to be improved across the women’s game due to the performance improvements that could be realised from this form of training.

Practitioners discussed some challenges players faced regarding the limited time to dedicate to gym-based training. Players often had additional responsibilities alongside their rugby careers, such as studying at university or having a second job due to low salaries. This is a common issue within women’s sports; a global review by FIFA [24] found that 36% of 736 female players surveyed were involved in formal education alongside their professional football careers. Twenty percent have full-time secondary employment, and 60% have a secondary job on a non-permanent contract. This time restriction means it is common for female players to miss certain aspects of their training, such as time in the gym:

*“…I skip some of the upper body stuff…I don’t have the time”.*

(P15)

This raised concerns with the practitioners about players’ upper body strength, with one practitioner remarking on players having difficulty performing upper body weight tasks:

*“They weren’t able to do 3 sets of 12 push ups on the floor and maintain form and scap(ula) control throughout, it’s a big work on”.*

(P13)

This could increase the players’ risk of injury, absorbing impact when falling to the floor or in collisions; this is particularly important for female athletes who physiologically have reduced muscle mass compared to their male counterparts [25]. Relating the focus of gym work to the players’ sport is essential to improving players’ engagement. However, the barrier they face is a lack of time to train due to poor salaries for most female athletes.

#### 3.1.3. Pitch Surfaces

This sub-theme relates to a resource barrier. Players and practitioners expressed concern that female players were more likely to play on lower-quality pitches than their male counterparts:

*“Women have been pushed onto different pitches. They haven’t been on the best pitch, and I think that does add to a lot of injuries”.*

(P6)

They felt this was due to the lack of availability of higher-quality pitches and the fact that they were not necessarily considered when assigning pitches. Players reported that they felt long-term male rugby players had preferential treatment for pitch allocation. A study by Petrie et al. [26] interviewed male, female and non-binary players to discuss their experiences of female access to rugby. Findings aligned with this study were that females felt they were allocated poorer pitches, which tended to be further away from the clubhouse. Players in this study felt these surfaces were harder, potentially predisposing them to injuries if they landed on a less forgiving surface, as one player remarked:

*“The pitch I did my shoulder on was genuinely like concrete. It’s rock solid… I do think that sometimes that’s not taken into consideration”*

(P3)

Players in this study felt there was likely to be an injury correlation with the poor pitch quality that they were repeatedly exposed to. Research within the men’s game has examined the link between injuries and artificial pitches [27], which found a high injury incidence in the lower limb. However, there is no research on the incidence of injuries related to pitch surfaces within women’s games. This lack of empirical research leaves female players devoid of evidence to support the feeling that inequitable pitch provision, compared to their male counterparts, contributes to injuries within the women’s game.

#### 3.1.4. Staff Turnover

The final sub-theme raises the issue of high staff turnover in the women’s game. Two practitioners and nine players discussed how they felt there was a high staff turnover in the women’s game, which regularly led to them having periods without medical support. Two players spoke about having no medical support during the offseason despite having a significant shoulder injury:

*“I did it at the worst possible time, when it came to like Physio care; it was the end of the season…I had no one to speak to”.*

(P7)

*“I didn’t speak to a Physio or see a Physio for 4 weeks and was told to stay in a sling”.*

(P6)

A similar issue was raised by several other players around the lack of consistency of medical staff or having multiple practitioners involved in their care throughout their injury rehabilitation. Players felt this caused an inconsistency in their rehabilitation:

*“Like every single camp, we had a different physio. So, I was continuously having these problems with my shoulder, but I was never seeing the same person, no continuity”.*

(P3)

This high turnover could be attributed to practitioners gaining experience in the women’s game and then successfully gaining better-paid employment in the men’s game [28]. Although we do not know the gender split of practitioners in the women’s game, female practitioners have been linked to a higher turnover than their male counterparts. Whilst there is a lack of evidence to explain why there is a high turnover, it can be suggested that family commitments, challenging work environments, and burnout all contribute [29]. Having consistent, well-qualified practitioners is important for developing professional relationships with athletes and, more broadly, with the team to help support injury prevention and management.

### 3.2. Theme 2: Different Viewpoints Between Players and Practitioners

This second theme shone a light on the relationship between athletes and practitioners, whereas theme 1 looked at the broader challenges within the women’s game. Practitioners and players had differing opinions on two key areas: First, when reporting injuries, players did not feel heard by the medical team, whereas practitioners felt players underreported. The second was around objective testing as part of the rehabilitation and RTP process, where players felt the testing did not correlate with their shoulder symptoms.

#### 3.2.1. Towards Injury Reporting: Players

The first sub-theme presents how players frequently did not feel heard by the medical staff when reporting a shoulder injury, felt they had to play on with their shoulder symptoms and the frustration they felt:

*“I was a bit frustrated about how I felt, like there was something wrong, and I was being told that there wasn’t anything wrong. So, I guess I felt… maybe not listened too fully”.*

(P2)

This communication issue was also evident in players’ reports, where they felt they were not given much clarity about their shoulder injury diagnosis. Players spoke about how they felt information, such as their scan results, was withheld, which caused them to mistrust the medical staff. One player remarked on the delay in receiving key information:

*“Lots of things weren’t communicated to me, like I find out [after] 16 weeks, [that] I’d actually broken it in two places instead of one”.*

(P4)

This quote echoes work by Coen et al. [30], who interviewed retired female athletes who discussed the challenges they faced having competed when their injuries had been dismissed, coupled with a strong sense that the medical team had not heard them. Furthermore, the feeling that they were being disbelieved and mistrusted in understanding their own bodies, despite feeling injured, demonstrates a need for a more person-centred approach from support staff in the women’s game.

It is important to recognise how some sub-cultures within sports normalise pain. In a systematic review by Soundy and Lim [31], dancers spoke about the perception of pain as something they felt was part of the role of being a dancer and associated it with working hard. The players shared a similar mindset:

*“I think it’s common for a lot of us, that we just talk about it. ‘Oh, it’s just your AC (Acromioclavicular joint). It’s fine, it’ll be sore, and then it’ll be fine’ and it’s very flippant”.*

(P7)

This can make it difficult for players to interpret whether the soreness they are feeling is something they should report, especially when they are unsure of the reception they may receive from the medical team, will their symptoms be taken seriously or ignored?

#### 3.2.2. Towards Injury Reporting: Practitioners

This second sub-theme looked at the different opinions practitioners had towards injury reporting. Practitioners felt frustrated with players not trusting them to tell them early or not telling them at all about their injuries. They felt that with the lack of early reporting, timely medical intervention could not be introduced, so players were more likely to exacerbate their injury and require a period of time off, as one explained:

*“We have a lot of under-reporting which leads to worse issues down the line… [if you] report a bad shoulder, you’ll be taken out of training, and that gives us, like these situations [where an injury is not reported], where we have a much worse injury”.*

(P13)

Practitioners expressed frustration that players did not report their shoulder injuries to avoid being withdrawn from training and match play. To minimise the risk of injuries worsening and requiring time off, practitioners are trying to educate players about timely injury reporting. One of the practitioners spoke about the work they are trying to do to educate players:

*“We’re trying to massively work on education around reporting injuries, and that reporting an injury doesn’t [necessarily] mean you’ll be withdrawn from training. It means that we can put in an intervention to try and keep you on the pitch, which is always a really tough one to get across”*

(P13)

A study by Rees et al. [32] found that younger and more inexperienced hockey players were more likely to underreport and attempt to self-manage an injury through painkillers. This contrasted with a more experienced hockey player who might be able to differentiate when they need to report an injury. The same study found that younger players feared that reporting an injury would result in them being perceived as weak, whilst more experienced players would underreport as they did not want to let the team down. They hid injuries before a major game or when joining a new club to avoid feeling they were making a negative impression. Improving communication between athletes and the medical team is therefore crucial and has been found to lower hamstring injury burden in elite football [33]. This is achieved by clarity over roles and responsibilities between players and the medical staff, with a more effective approach to load management.

#### 3.2.3. Objective Testing: Players

This third sub-theme focused on the players’ perspectives towards objective testing. Players discussed how they felt the objective tests practitioners used for shoulder injuries were not specific to or functional for the sport, and the results did not correlate with their symptoms. One player said:

*“I’m strong in all my (testing) positions. But it doesn’t feel strong if that makes sense so, like it feels weak”.*

(P1)

They felt they would be told their shoulder was strong based on the test results, but they did not feel they had strength parity in the gym, and their shoulder felt weak when tackling on the pitch. Consequently, players lacked trust in the tests and did not understand why they were used as a marker to return them to rugby, as one player remarked:

*“I don’t trust this test, but this is what’s stopping me from playing any form of Rugby”*

(P3)

Female rugby players may not trust shoulder objective markers because these metrics often fail to account for gender-specific, biomechanical differences and injury patterns. Additionally, a lack of representation in research and standardised benchmarks based on male data can lead to scepticism about their relevance and accuracy.

#### 3.2.4. Objective Testing: Practitioners

The fourth sub-theme was around practitioners’ perspectives towards objective testing. Practitioners in this study and more widely acknowledge that objective testing is a complex process in elite sport and that it is a challenge to ascertain normative values in the elite population that result in valid and reliable testing [4]. One practitioner spoke about trying to gain more data points in pre-season:

*“We are trying to build it with pre-season data, but there’s really not much out there… It’s difficult, I think, for women in general. It is definitely better than it was 5 years ago”*

(P17)

Establishing normative values is steadily progressing, but testing data on females is still lacking. As one practitioner noted, the lack of resources and the requirement for portable testing equipment can affect the reliability and validity of available testing methods:

*“It’s less valid and probably less unified across the League in terms of what objective markers people use for the upper limb, in comparison with the lower limb”*

(P17)

There is no clear information on what data are needed to form criteria to clear an athlete following a glenohumeral joint stabilisation [34]. In a systematic review by Ciccotti et al. [35], time was the primary measure across 75% of the studies, deciding whether the patient was fit to return to sport after 6 months post-surgical stabilisation. However, this does not provide objective data that the shoulder and athlete could return to play post-injury successfully. Medical and S&C practitioners’ decision-making process in returning an athlete to play is complicated with the threat of recurrence, medical litigation, and implications for their health and performance. Selecting objective tests needs to be a collaborative approach between players and practitioners in deciding the key areas they are trying to test and the impact of the results in the decision-making process.

### 3.3. Theme 3: Threat to Identity

This third theme differs from the previous two as it was raised only by the players following a significant injury and was emotive for the players to discuss. The theme of threat to identity emerged when players discussed feelings of isolation, whether that was from continuing to play despite being injured, due to fear of missing out on selection, or not wanting to go back through rehabilitation, which they felt was unsuccessful previously. The isolation they felt and their lack of certainty about whether they were going to be able to return to their career as rugby players, were emotive areas for them to discuss.

#### 3.3.1. Getting on with It

This first sub-theme was about how players felt they had to “get on with it”. Two players discussed their long-term recurring shoulder injuries and stopped reporting their symptoms as they did not want to miss any more fixtures. With one player reflecting back on the experience:

*“I just played on because I was like… I know it sounds really bad now, but I can’t be out for this again. I’ve been out for so long”.*

(P1)

Players had missed a significant period already, and they were keen to avoid the threat of being out again and the further impact it would have on their career, mental and physical health. This sub-theme is linked closely with the fourth theme, which is about returning to play and their confidence, and the second theme, injury reporting. Participant 1 spoke about the fear of being dropped:

*“I didn’t want to get dropped, but I didn’t really think it was that significant until it came out(dislocated). Obviously, like the symptoms I got with it, I should have gone that’s actually not normal”.*

(P1)

Squad selection was frequently discussed and how this weighed heavily on them and their decision making around when to ‘get on with it’. The attitude of ‘getting on with it’ at all costs is often found in sport [36]. Developing trust and communication with the medical staff is an important aspect of managing this and requires a culture of care, adopting a big picture perspective to avoid a short-term, results-driven approach.

#### 3.3.2. Rehab Isolation

In the second sub-theme, players discussed the difficulty of being isolated from the team during rehabilitation and how no longer operating on the same schedule and needing to juggle rehab with team commitments had a detrimental impact. One player suggested that it would have helped her feel more a part of the team if she could have performed her running conditioning alongside the team rather than separately:

*“I guess it’s just really isolating, being injured in a team sport…rather than making me run in my own time, I could run alongside the team, or, still be at training and still jump in and out. But just being around the team is nice”.*

(P1)

This can exacerbate the feeling of being isolated during the rehabilitation process. Several players discussed how they were made to do their running and gym work separately from the rest of the team, being taken away from their usual environment. This social isolation can add to the loss of athletic identity [37], which in turn influences their RTP, where having a support network, especially one that includes peers who have undergone a similar injury, can positively impact the athlete by providing them with reassurance and help in managing expectations around the rehabilitation and RTP process [38]. This was reflected by three of the players in this study, with Participant 2 saying:

*“It was nice to speak to someone that knew what I was sort of going through, and it was nice to see someone coming out the other side”*

(P2)

Two players also spoke about having regular touchpoints with their S&C coaches and medical staff, which helped them feel less lonely and found this helped with their rehab process:

*“It was a really good dynamic like. I was very lucky to have them in my rehab. It just made it much smoother, and it was easier for me”*

(P2)

Having a support network around the injured player assisted them with the difficulties they were navigating psychologically around their injury, alongside the tissue pathology.

#### 3.3.3. Threat of Career-Ending

The final sub-theme was an emotive topic area for players to discuss: the threat to their career. Two players spoke of their adverse interactions with surgeons, with one player sharing how the surgeon told her she was not built for rugby and that she should stop playing, while another said she felt time pressured to decide whether to proceed with surgery and that she was not seen as a person.

*“I’ll be able to sell the beds to other NHS patients. And I was like, Sell the beds. I was like Jesus, I’m a person here, this is my career that we’re talking about”*

(P4)

The interpersonal skills of surgeons have been found in other research to be an area for further development. Van Iserel et al. [39] found that patients felt there was a lack of empathy expressed by their surgeons and a lack of awareness about how the shoulder surgery would impact their lives. The relationship between the athlete and the medical team during the rehabilitation process is essential. Yet, some patients described their care as a “dictatorship” with a lack of collaboration to understand what the patient wanted to achieve. This fostered a relationship of uncertainty and fear around their shoulder injury [38]. Participant 3 spoke about how trust was affected:

*“You trust the professionals. But you probably need to question things a bit more than I maybe did question at the time”.*

(P3)

There was an expectation of trust due to being a medical professional, but this did not necessarily correlate with the relationships built with the player.

Another player investigated alternative career options with other sports following a discussion between multiple medical team members, which left her feeling there was no return to rugby following her shoulder injury. She discussed how her shoulder injury defined a significant portion of her career and held her back from playing opportunities:

*“I definitely feel like it really defined a couple of years in my Rugby career….I felt like it was definitely holding me back”*

(P2)

This uncertainty about whether they would return from their injury during their rehabilitation affected their confidence in the process, contributing further to the fear of returning to sport.

### 3.4. Theme 4: Return to Play

The fourth theme focused on the final stages of the rehabilitation journey, the transition from injury back into contact and match play, and how certain things, such as strapping and peer support, gave them confidence. However, there were certain positions in which their shoulders still felt vulnerable, and difficulty in reading the game after being out for a prolonged period affected their confidence.

#### 3.4.1. Confidence

This first sub-theme focused on confidence, and players discussed several helpful strategies that promoted confidence in the RTP process following a shoulder injury. Two spoke about having peer support from other players who had also had shoulder injuries and had successfully returned to play; this provided a real-world example of success and provided the opportunity to speak with like-minded players about the challenges they had experienced. Participant 4 talked about the support it gave her:

*“Being able to speak to someone that had gone through it, and was like someone that I trusted. And I’d seen her get back to Rugby as well was something that like was really helpful for me”*

(P4)

This support can also come from practitioners who had helped players feel supported during rehabilitation. One player spoke about the positive impact they had for her:

*“I just had a really amazing team behind me”*

(P3)

Frequent feedback between practitioners and players enhances performance and development opportunities [40], which would assist the players in feeling supported and gaining confidence in their shoulder to return to play.

Multiple players discussed how strapping gave them confidence in their shoulders; but they did say it also became habitual, as one player discussed:

*“I feel like it kind of puts my mind at ease like there is something there, and it’s not just fully like just the shoulder”*

(P7)

The motivation for this could be that, despite being cleared to play, they could still feel overly protective of their injured shoulder [41]. Although research into the efficacy of strapping is inconclusive, that it can enable athletes to feel more confident is widely recognised [42].

Practitioners discussed various ways of building a player’s confidence in their shoulders, such as gradual exposure to contact and having multiple team members check in on how the player feels with their RTP. One practitioner discussed how they approached this:

*“It’s about ensuring you’ve had that conversation to make sure they are comfortable, and they haven’t got any outstanding concerns or issues that we can maybe broach with them before that return to play”*

(P18)

Having coaches involved in the RTP to develop tackle technique links back to the first theme around establishing safe, effective practice.

#### 3.4.2. Lacking Confidence

The final sub-theme was about things that negatively affected players’ confidence. One of them was a feeling of vulnerability from having their arm out in an abducted position in the gym or on the field of play, which increased the fear of injury. This was highlighted by participant 1:

*“I struggled sort of being like in a vulnerable position…being in positions that aren’t like as strong”*

(P1)

The shoulder position they are referring to was being abducted 90 degrees and externally rotated 90 degrees. This is the typical position the shoulder would be in for a traditional tackle technique on another player, and is associated with a high prevalence of shoulder injury [43]. This links back to the need for female players to have high-quality coaching, to assist with building players’ safety, ability and confidence in tackling. Also, avoiding using the word “vulnerable” and the association of the shoulder being at risk could enhance a player’s confidence in their shoulder.

When returning to match play, players discussed how they found it challenging to transition from structured drills to unstructured play. They felt that a large gulf between the two made it difficult to understand how to position themselves to perform. One participant gave an example of this:

*“It very much was a confidence thing like I had a mental block. I couldn’t really get around on pitch; I was all over the place. It was pretty much my first time playing, so in the drills, I was fine, and when it got to the pitch, I was like, what is going on?”*

(P2)

Players found that, given the length of time they had been out injured, it was more challenging to read and understand the chaotic environment of matches, which affected their confidence on the pitch and their performance. Utilising frameworks such as the ‘control–chaos continuum’ [44], which involves gradual exposure from high control to more unpredictable and varied movement patterns that players would usually display within a match situation, would assist the players in feeling more confident about their return and their rugby performance.

### 3.5. Theoretical Framework

Reflecting on the data and the four themes that emerged, there was a common trend with practitioners and players discussing how they felt, whether that was feeling listened to, trusted or belonging. This correlates with the feminist care theory by Noddings [45]. This discusses the importance of caregivers practising and reflecting on the act of caring and the shared role between the care provider and receiver. The focus is more on the caring process than the results. There are two different aspects to care: one is “caring about”, and the other is “caring for”. “Caring about” is seen, for example, in caring for people starving in a third-world nation; they care about them and are thereby motivated to donate money to a cause to support them [46]. On the other hand, “caring for” is seen more in the acts of caring, doing something for the person and trying to understand what that person is feeling and experiencing while putting one’s interests aside [47].

These concepts of care are evident throughout this research. When the players discussed their negative interactions with the surgeons, the focus of the care was on the end result of repairing their shoulder issue, not on the delivery of the message and understanding of the impact this surgery had on the player and their broader career. The process of caring for the athlete was absent.

The lack of communication between practitioners and players, with players feeling key parts of their medical scans and information were being withheld, is an example of a lack of “engrossment”. It could be seen that the athlete is not being “cared for”, as the practitioner does not understand the athlete’s perspective and how the athlete might feel and want to know this information. This can be difficult due to the environment the practitioner is in and the external influences and pressure they might be facing, such as a coach demanding a quick return for a key player [48]. The practitioner is unable to put their own interests aside to understand and care for the athletes’ wants and needs, and instead focus on the end goal. The drive to improve tackle technique and strength training to become more female-specific should not solely focus on female physiology, such as menstrual health and breast health/injury, but also on the delivery and process that enables the player to feel cared for.

There are multiple examples where practitioners have modelled “caring for”, and the athlete has reflected this back. This is seen particularly in the fourth theme, RTP, and the confidence sub-theme. The players spoke about how they felt supported by the practitioners, enabling a smoother rehabilitation process and opportunities to discuss their fears and hesitations about returning to play. The role of peer support from other players enabled the injured players to voice their concerns and feel listened to about the journey and process they are going through.

Collaborating with players and understanding the players’ needs and experiences would help address the issues highlighted around the objective tests and consideration of tests that instil confidence for both the player and practitioner.

### 3.6. Limitations

There were several limitations to this study. One is that all the participants were English and of white ethnicity, which does not reflect the broader women’s rugby union community. The participants who took part likely did so due to their interest, experience of shoulder injuries, and willingness to discuss these, which may not represent the opinions of those from the wider game.

Demonstrating rigour in qualitative research is a thorny issue [49]; however, we make no apology for the inevitable subjectivity inherent during data analysis. The methodology that was selected could have limited the interpretation of the results. Although the COREQ was used as a checklist, we did not use a priori theoretical understanding to interpret and acknowledge. Nevertheless, it should be noted that the lead researcher, being primarily known as a physiotherapist rather than a researcher, could have influenced how participants responded to the interview questions.

### 3.7. Clinical Implications

Applying Noddings’ theory of care reveals that truly supporting female athletes extends beyond technical competence; it requires empathic, consistent, and collaborative relationships. This includes equitable access to high-quality facilities, tailored education for coaches and practitioners, and rehabilitation processes prioritising psychological safety, continuity of care, and confidence building. By embedding these care-based principles into practice, practitioners can move from a model of ‘caring about’ performance outcomes to genuinely ‘caring for’ the individual, enhancing both recovery and long-term engagement in the sport.

Enhancing care for elite female rugby players necessitates a coordinated, evidence-informed strategy. Equal access to high-quality pitch surfaces and staff, comparable to those provided in the men’s game, may be a critical yet straightforward strategy that could reduce injury risk and promote performance equity. Practitioner and coaching education development that focuses on issues pertinent to the female athlete would help address the knowledge gap. Furthermore, effective, ongoing communication between the multidisciplinary team (MDT) and the athlete is essential to optimise injury reporting, rehabilitation adherence, and long-term athlete well-being.

## 4. Conclusions

This study offers critical insight into the rehabilitation experiences of elite female rugby union players following shoulder injury, capturing both athlete and practitioner perspectives. The findings highlight the need for an athlete-centred, contextually sensitive approach to rehabilitation and return to play, where psychosocial and environmental factors are considered as vital as physiological recovery. The disconnect between players and practitioners—especially around injury reporting, communication, and the interpretation of objective testing—demonstrates the importance of developing shared frameworks that incorporate players’ lived experiences, emotional responses, and sport-specific performance demands.

Future research should include more diverse populations and explore the implementation of care-informed practices within high-performance settings. A key direction for development is the creation of a practical toolkit underpinned by the care theory framework. This would provide clinicians, coaches, and support staff with structured guidance to deliver holistic, empathetic, and collaborative care throughout the rehabilitation and RTP process, ultimately improving outcomes for female athletes.

## Figures and Tables

**Table 1:** Summary table of the 4 main themes, by which participants were generated, and sub-themes with corresponding quotes.

Theme	Sub-Theme	Frequency of Associated Data Extracted	Athlete and/or Practitioner Generated	Quote and Participant Number Related to (P)
Growth of women’s game	Tackle technique	17	6 practitioners and 3 athletes	“As they fatigue I think the tackle technique deteriorates. So that’s like a fitness thing” (P20)
	Strength training	20	9 Practitioners and 5 athletes	“really good wins when it came to just general high level understanding of why we would do an upper body lift from a strength perspective” (P15)
	Pitch Surfaces	5	4 Practitioners and 5 athletes	“I also wonder about the surfaces, because it seems to be a lot more artificial surfaces in the women’s game” (P16)
	Staff turnover:	13	2 Practitioners and 9 Athletes	“I sort of got passed over to someone else who didn’t really know anything about me or my shoulder// there was like a bit of time where I didn’t really have any one. I was just sort of doing my own thing”. (P1)
Different viewpoints between players and practitioners	Towards injury Reporting: Players	9	11 Athletes	“It was quite unclear of like what I was when I would be back and like what I’d actually done” (P2)
	Towards injury Reporting: Practitioner	13	5 Practitioners	“We’re not getting necessarily accurate reporting, and then they get to the point where they have really unstable shoulders” (P17)
	Objective testing: Players	17	6 Athletes	“I think something that was really helpful for me coming back was when we were more looking at markers in the gym” (P3)
	Objective testing: Practitioner	16	8 Practitioners	“Using the objective data from the strength testing to give me confidence. So as a practitioner is the shoulder is there good output from the shoulder? Yes or no?” (P20)
Threat to identity	Getting on with it	15	7 Athletes	“ It was like first session back, and I was just thrown in right at the deep end. I also knew this was my only chance to put my hand up for selection. I just needed to get on with it” (P4)
	Rehab isolation	9	11 Athletes	“It’s quite like lonely. You’re doing it all yourself. So it’s nice to have like a lot of points of contact with like Physio’s, S&Cs. And I did find that really helpful” (P2)
	Threat of career ending	13	4 Athletes	“I think the physio at the time was just being like trying to give me a picture of, like what the worst case scenario could be. I went home and email British cycling” (P3)
Return to play	Confidence	25	9 Practitioners and 8 Athletes	“I definitely feel more like confident when it’s strapped. But I do also think strapping can become a bit of a habit” (P4)
	Lacking confidence	29	2 Practitioners and 8 Athletes	“I did sort of know it wasn’t quite right. I still couldn’t reach really high up and be like pushed out in like vulnerable positions that just didn’t feel very strong”. (P1)

## Data Availability

Data is unavailable to share to protect the privacy of the participants.

## Supplementary Materials

The following supporting information can be downloaded at: https://www.mdpi.com/article/10.3390/sports13060166/s1, Table S1: Theme generation.

## Abbreviations

The following abbreviations are used in this manuscript:

RTP	Return to Play
RTS	Return to Sport

## Appendix A. Semi-Structured Interview Question Guide

Players, who have incurred a significant shoulder injury:

- What was your shoulder injury mechanism?
- What do you deem as a successful return to play following a shoulder injury?
- How did you feel during the injury period?
- How do you feel that the collaboration worked between you, the coach and the person or persons who were responsible for your rehabilitation?
- What do you feel are the key components for assessing if you are ready to return to
- Can you tell us about how you felt when you were going to return to sports again? Was there anything that you found difficult, and if so, what?
- Following your return from your injury, were there any changes to your programme or did you undertake any specific shoulder work outside of your regular programming?
- Can you give examples of something that you thought worked well regarding the help you received in connection with being injured?
- Can you give examples of something that you think could be improved regarding the help you received in connection with your injury?
- How did you experience/think it was to be injured and have difficulty participating or not being able to participate in training/competition?
- Is there anything else you want to add that you think is important about this area and that we have not talked about?

The medical team and strength and conditioning coaches:

- What do you think is the most common injury mechanism for the shoulder?
- What do you deem as a successful return to play following a shoulder injury?
- Which staff members do you think are important to be involved in the return to play? Who do you think should lead this? How have you found working with them and the collaboration piece?
- What do you feel are the key components for assessing if they are ready to return to play following a shoulder injury?
- How do you think this can be objectively assessed?
- When do you decide if an athlete is not able to return to play following a shoulder injury?
- What do you think are some of the challenges for these athletes?
- What do you think is missing from the research or unknown in the RTP of these athletes?

Focus group questions with players who have not incurred a significant shoulder injury:

- What do you consider to be a shoulder injury?
- Pain—What is a good pain/bad pain?
- When does pain start to affect performance?
- How would you define a shoulder injury?
- What factors influence the occurrence of a shoulder injury?
- What is the most likely mechanism for a shoulder injury in collision-based sports?
- How would you define shoulder injury prevention?
- Who do you think is responsible for shoulder injury prevention?
- What shoulder injury prevention strategies have you applied and why?
- How do you choose shoulder injury prevention strategies?
- Which factors make shoulder injury prevention more difficult?
